# A novel, putatively null, *FGD1* variant leading to Aarskog-Scott syndrome in a family from UAE

**DOI:** 10.1186/s12887-017-0781-4

**Published:** 2017-01-19

**Authors:** Abdul Rezzak Hamzeh, Fatima Saif, Pratibha Nair, Asma Jassim Binjab, Madiha Mohamed, Mahmoud Taleb Al-Ali, Fatma Bastaki

**Affiliations:** 1 Centre for Arab Genomic Studies, P.O. Box 22252, Dubai, United Arab Emirates; 2 Pediatric Department, Latifa Hospital, Dubai Health Authority, Dubai, United Arab Emirates; 3 Pediatrics and Neonatology Department, Dubai Hospital, Dubai Health Authority, Dubai, United Arab Emirates

**Keywords:** Faciogenital dysplasia, X-linked Aarskog syndrome, Frameshift deletion, Emirati, Consanguineous

## Abstract

**Background:**

The X-linked condition “Aarskog-Scott syndrome (AAS)” causes a characteristic combination of short stature, facial, genital and skeletal anomalies. Studies elucidated a causative link between AAS and mutations in the FGD1 gene, which encodes a Rho/Rac guanine exchange factor. FGD1 is involved in regulating signaling pathways that control cytoskeleton organization and embryogenesis.

**Case presentation:**

FGD1 was studied in an Emirati family with two cases of AAS using PCR amplification and direct sequencing of the entire coding region of the gene. Various in silico tools were also used to predict the functional consequences of FGD1 mutations. In the reported family, two brothers harbor a novel hemizygous mutation in FGD1 c.53del (p.Pro18Argfs*106) for which the mother is heterozygous. This frameshift deletion, being close to N-terminus of FGD1, is predicted to shift the reading frame in a way that it translates to 105 erroneous amino acids followed by a premature stop codon at position 106. Full molecular and clinical accounts about the variant are given so as to expand molecular and phenotypical knowledge about this disorder.

**Conclusions:**

A novel variant in FGD1 was found in an Emirati family with two brothers suffering from AAS. The variant is predicted to be a null mutation, and this is the first report of its kind from the United Arab Emirates.

**Electronic supplementary material:**

The online version of this article (doi:10.1186/s12887-017-0781-4) contains supplementary material, which is available to authorized users.

## Background

Aarskog-Scott syndrome (AAS) or faciogenital dysplasia (FGD) is an X-linked syndrome with a recessive mode of inheritance (OMIM #305400). This condition is characterized by a distinguishing combination of short stature, facial, genital and skeletal anomalies. The latter include hypertelorism, short nose, brachydactyly, syndactyly and shawl scrotum. Additional features include; mental retardation, joint hyperextensibility, ptosis and inguinal/umbilical hernia. Importantly, clinical presentations of patients vary to a great extent, which impedes reaching a clear-cut diagnosis. This is further exacerbated by the difficulty to establish the carrier status in asymptomatic females, in whom these symptoms may become less apparent with age. In these cases as well as in instances when the condition is confused with other syndromes due to symptoms overlap, the diagnosis is made depending on molecular characterization of the patient and her/his family [1].

The *FGD1* gene was mapped to Xq11.22, on the short arm of the X chromosome. Spanning over 51 kb, FGD1 encompasses 18 exons and encodes a protein that is 961 amino acids long. The latter protein is a guanine nucleotide exchange factor for the Rho GTPase cell division cycle 42 (CDC42). FGD1 belongs to the DBL family of proteins and is considered essential for normal embryogenesis in mammals; with an important role in skeletal development and morphogenesis. Additionally, FGD1 is a potential regulator of extracellular matrix remodeling, which is strongly connected to its roles in cytoskeletal organization [2, 3].

Wild-Type FGD1 is composed of several functional domains. The central region of FGD1 comprises a DBL homology (DH) domain and a pleckstrin homology (PH) domain, and these two mediate the exchange of CDC42-bound GDP for GTP. N-terminally, there is a proline-rich domain containing two putative Src homology 3 (SH3)-binding sites. Towards the C-terminus, there are two domains; a FYVE domain followed by a second PH domain. Several types of mutations affecting these domains have been previously reported in AAS patients, and the majority of missense point mutations affect the central catalytic domains. Additionally, nonsense and frameshift mutations were reported in the context of this condition. Importantly, deletions, inversions and duplications were observed to a lesser extent and most mutations are unique within families with no obvious genotype–phenotype correlation in patients [4].

**Fig. 1:**
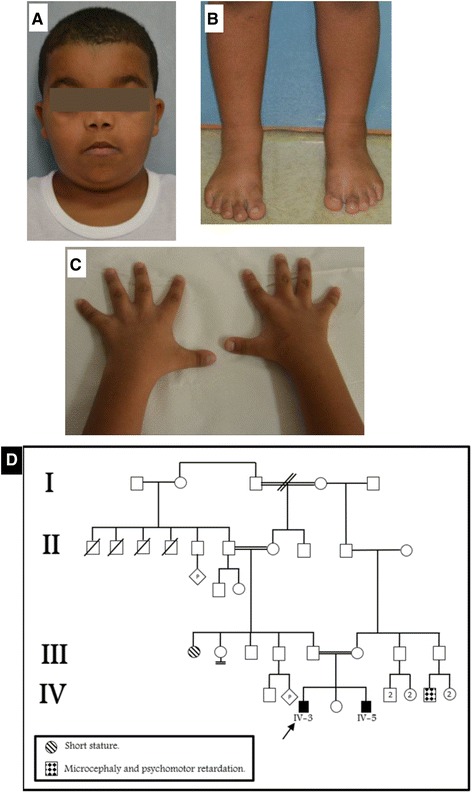
Clinical features of the proband (the elder brother) with Aarskog-Scott syndrome: Panel **a** illustrates facial dysmorphic features. Panels **b** and **c** illustrate skeletal abnormalities in the patient. Panel **d** shows a pedigree of the affected family

We present here an Emirati family with two siblings suffering from AAS (Fig. 1d) due to a novel hemizygous mutation in *FGD1*. A full account of the clinical features in the affected brothers as well as their previously-unreported molecular lesion is given in this study.

## Case presentation

The proband (IV-3) was a 3-year-old boy referred to the genetic clinic with developmental delay, speech delay and dysmorphic features. He was born at term via Ceasarian section due to fetal distress with a birth weight of 2.5 Kg (<10th centile). Physical examination revealed an aggressive and hyperactive boy. Height at 89.5 cm was at the 3rd centile, while weight and head circumference at 13.6 Kg and 48.5 cm, respectively, were at the 25th centile. Dysmorphic facial features that were recorded included a broad forehead with a widow’s peak, bilateral partial ptosis, blepharophimosis, hypertelorism, epicanthic folds, down slanting palpebral fissures, broad nasal bridge, anteverted nostrils, short columella, everted lips, low set malformed ears with thickened helix, short philtrum, micrognathia, short neck, and a high arched palate (Fig. 1a). Nipples were low set bilaterally, and widely spaced. Pectus carinatum with excavatum was noticed, along with brachydactyly, bilateral fifth finger clinodactyly, bilateral partial syndactyly of the second and third toes, interdigital webbing, and single palmar creases (Fig. 1b and c). Sleep disturbance was reported. All other investigations were normal, and karyotype analysis revealed a 46, XY karyotype. At follow-up examination at 7 years, he was found to have proportionate borderline short stature (height 100 cm; 2nd-50th centile). He was obese with a Body Mass Index of 21.8 Kg/m2 (98th centile). Developmental delay was noticed in that he was unable to throw a ball or button his clothes, did not know his birthday, and needed help to maintain self-hygiene. He was found to have a left undescended testis, for which orchidopexy was scheduled. He continued to be hyperactive and had poor control on his attention. He was attending normal stream school with acceptable performance.

**Fig. 2:**
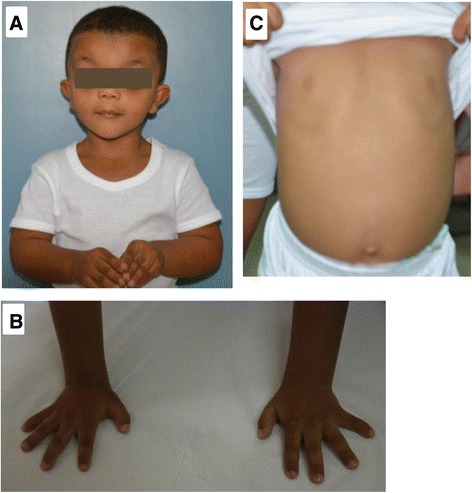
Clinical features of the younger brother with Aarskog-Scott syndrome: Panel **a** illustrates facial dysmorphic features. Panel **b** shows skeletal abnormalities in the patient. Panel **c** shows the chest deformity and widely spaced nipples

His younger brother (IV-5) presented at 3 years of age with similar features of short stature, hyperactivity, developmental delay, and dysmorphic features. His weight stayed below the 3rd centile since his birth, and weight was below the 10th centile. Like his brother, he had a broad forehead with a widow’s peak, bilateral partial ptosis, blepharophimosis, telecanthus, hypertelorism, epicanthic folds, down slanting palpebral fissures, broad nasal bridge, short nose, anteverted nostrils, short philtrum, malformed ears with thickened helix, and a short neck (Fig. 2a). Detailed ophthalmologic examination revealed overacting frontalis muscle, bilaterally poor levator muscle function, and poor Bells phenomenon. He was investigated at the ENT clinic for snoring, and found to have mild right eardrum retraction and a long uvula. At the age of 2 ¾ years, he was unable to throw a ball, walk backwards or to the sides, tiptoe, perform a tandem gait, stand on one leg, draw a line, or bead a thread. He could not differentiate between night and day. He was aggressive and hyperactive. His hands were broad and short, with brachydactyly, and bilateral fifth finger clinodactyly (Fig. 2b). Like his brother, he had low set, widely spaced nipples (Fig. 2c).

The patients’ parents were first half cousins. The mother has a short stature (HT 152.5 cm) with widow’s peak and hypertelorism. One of the patients’ maternal cousins was a 4-year old with microcephaly and developmental delay, while four of their paternal uncles were reported to have died in their adolescence due to unknown causes. A paternal aunt was reported to have a short stature.

**Fig. 3:**
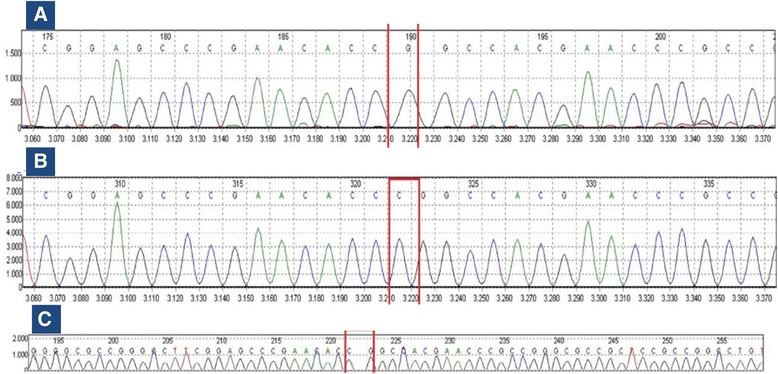
Sequence chromatograms showing the novel *FGD1* variant in a hemizygous state in the two brothers; Panels **a** and **b** illustrating a reference chromatogram and the younger brother’s DNA respectively. Panel **c** shows relevant DNA sequences from the proband

The two brothers were found to harbor the novel *FGD1* variant c.53del (p.Pro18Argfs*106) in a hemizygous state (Fig. 3) while the same variant was detected in heterozygosity in the mother. *FGD1* sequencing did not reveal other potential candidates for causality of the abovementioned symptoms in the mother and her sons. The variant was not reported in any of the databases; dbSNP, 1,000-genomes, Leiden Open Variation Database, EVS and ExAC. On the other hand, the variant was not found in the GalaxC™ Allele Frequency Database which contains >2.5 million unique Middle Eastern pathogenic mutations and variants. The mutation c.53del leads to a frameshift that produces “out of frame” translation of 105 erroneous amino acids followed by a premature stop codon at position 106. ExPASy translational tool was used to obtain the above-mentioned translation.

## Conclusions

This study reports a novel mutation in *FGD1* in an Emirati family with two affected brothers, and these are the first fully characterized cases of Aarskog-Scott Syndrome from the country. Due to the considerable variability of AAS phenotypic features, clinical diagnosis depends on fulfilling a number of primary and secondary criteria as per Teebi *et al.*1993 [5]. The affected brothers presented with a number of the primary features of AAS including; hypertelorism, short stature, short nose, anteverted nostrils, interdigital webbing and bilateral fifth finger clinodactyly. Additionally, some of the “secondary criteria” were also present; namely, widow’s peak, ptosis, downward slant of palpebral fissures and cryptorchidism. Importantly, certain additional features were well noticeable in the two patients, especially in terms of hyperactivity and aggressive behavior. Categorizing clinical features in the abovementioned style gives the rather illusive impression of the possibility of reaching a definitive diagnosis through clinical evaluation alone. Unfortunately, this is not the case because actual patients usually present with combinations of symptoms that appear frequently in numerous other syndromes. Moreover, there are cases of clinically-evident AAS which could not be attributed to mutations in *FGD1*. As a matter of fact, some researchers argue that the majority of AAS cases are still to be characterized molecularly, with *FGD1* mutations being established in a mere 20% of the cases [6]. Consequently, cases are increasingly being subjected to molecular analysis in order to determine the underlying causative variant.

This p.Pro18Argfs*106 change is extremely close to the N-terminus; therefore it is likely to compromise all functional domains of the protein. This scenario is corroborated by the score obtained by CADD [7], which yielded a PHRED scaled score of 21. The latter score indicates the variant is predicted to be among the 1% most deleterious substitutions that can happen to the human genome. There are numerous published accounts of *FGD1* mutations that affect all the domains of the protein, however only one null mutation was uncovered [8]. The here-reported variant probably leads to total loss of *FGD1*; however, phenotypical comparisons between the two mutations do not provide any exclusive common findings especially in relation to behavioral symptoms. This is hardly surprising since *FGD1* mutations do not show clear genotypic/phenotypic correlations for mutations affecting the same domain, or even in the cases with the same mutation affecting different patients [9].

Both patients were found to suffer developmental delay, aggressive behavior and hyperactivity. Various manifestations affecting cognition and behavior were reported before in patients with *FGD1* mutations, in which some elements of Attention Deficit Hyperactivity Disorder (ADHD) can be present. The common feature, however, among patients with *FGD1* mutations is the absence of severe mental retardation [1, 10, 11]. In the same vein, obesity was observed in one of the patients as is the case with some AAS patients [9], however the causal link between AAS and obesity awaits better confirmatory findings before including obesity in AAS diagnostic criteria.

In addition to the novelty of this variant, it is the first to be reported from the United Arab Emirates. Two previous studies have reported different *FGD1* mutations in Arabs [12, 13]. It seems that the majority of the reported mutations in *FGD1* are novel ones while recurrent mutations are fewer [1, 9]. In light of varying clinical features of this syndrome and its overlap with other conditions, it is important that the medical literature is kept updated about novel mutations emerging in different ethnic backgrounds.

## Additional file

Additional file 1: Methods. (DOCX 14 kb)

## Abbreviations

AAS: Aarskog-scott syndrome
CADD: Combined annotation dependent depletion
CDC42: Cell division cycle 42
DH: DBL homology
ENT: Ear, nose and throat
PCR: Polymerase chain reaction
PH: Pleckstrin homology
SH3: Src homology 3
